# PTHR1 in osteosarcoma: Specific molecular mechanisms and comprehensive functional perspective

**DOI:** 10.1111/jcmm.16420

**Published:** 2021-03-06

**Authors:** Chaonan Sun, Shenglong Li

**Affiliations:** ^1^ Department of Radiation Oncology Cancer Hospital of China Medical University Liaoning Cancer Hospital & Institute Shenyang China; ^2^ Department of Bone and Soft Tissue Tumor Surgery Cancer Hospital of China Medical University Liaoning Cancer Hospital & Institute Shenyang China; ^3^ Department of Tissue Engineering Center of 3D Printing & Organ Manufacturing School of Fundamental Sciences China Medical University (CMU) Shenyang China

**Keywords:** oncogene, osteosarcoma, prognosis, PTHR1, targeted therapy

## Abstract

Osteosarcoma occurs largely in children and adolescents and is the most common primary malignant tumour of bone. Although surgical advances and neoadjuvant chemotherapy have made great strides in recent years, rates of local recurrence and lung metastasis remain high, with a plateau in overall survival during the past decade. It is thus urgent to explore the pathogenesis of osteosarcoma and identify potential therapeutic targets. Parathyroid hormone receptor 1 (PTHR1) belongs to the broad family of G protein–coupled receptors, binding both parathyroid hormone (PTH) and parathyroid hormone–related peptide (PTHrP, a paracrine factor). Previous studies have shown that in tissues and cells of osteosarcoma, expression of PTHR1 is markedly increased, correlating with aggressive biologic behaviour and a poor prognosis. PTHR1 expression also correlates closely with epigenetic regulation, transcriptional regulation, post‐translational modification and protein interaction. Herein, we have summarized the latest research on the role played by PTHR1 in progression of osteosarcoma, assessing its clinical utility as a novel biomarker and its therapeutic ramifications.

## 1 INTRODUCTION

Osteosarcoma is a common and often rapidly progressing primary malignant tumour of adolescents and children. It tends to arise near the ends of limbs and long bones but may involve the iliac bone, spine and other skeletal parts. There is a proclivity for distant metastasis as well, and the generally low long‐term survival rate of such patients reflects its high degree of malignancy.^1^, ^2^, ^3^ Surgery is the chief means of early treatment for osteosarcoma^4^, ^5^ (Table 1). However, this method has two clear disadvantages, namely the trauma entailed and the less than encouraging prospects of long‐term survival.^6^ The 5‐year survival rate in patients treated exclusively by surgery is quite low, owing to pulmonary metastases, prognostic factors and matters of therapeutic compliance.^7^, ^8^

**TABLE 1:** Potential therapeutic approaches of osteosarcoma

Approach	Related drugs	Related gene
Surgery		
Tumour resection	/	/
Limb function reconstruction	/	/
Chemotherapy	MTX, ADM,	/
	DDP, IFO	/
Radiotherapy		
Three‐dimensional conformal radiation therapy (3D‐CRT) Intensity‐modulated radiation therapy (IMRT) TomoTherapy (TOMO)	/	/
Molecular targeted therapy	/	Ezrin, HER2, telomerase
Immunity therapy		
DC therapy	/	/
Cytokine therapy	/	/
CAR‐T cell immunotherapy	/	/
Immune checkpoint block therapy	/	PD‐1/PD‐L1
Gene therapy	/	p53, p16, p21 and Rb
Embolization therapy		
Selective arterial embolization	/	/
Transcatheter arterial chemoembolization	/	/
Radiofrequency ablation therapy		
High intensity energy focused ablation	/	/
Radiofrequency ablation	/	/
Cryoablation	/	/
Microwave ablation	/	/
Stem cell therapy	/	HH, NOTCH,
		Wnt/p‐catenin and MAP

More recently, molecular mechanisms fuelling the development and progression of osteosarcoma, particularly LncRNA DANCR promotion of ROCK1‐mediated proliferation and metastasis (via decoying of miR‐335‐5p and miR‐1972) or expression patterns of programmed death proteins (PD‐L1, PD‐L2 and PD‐1), have provided a crux for therapeutic targeting.^9^, ^10^, ^11^ Still, an optimal approach to routine chemotherapy has yet to be devised. Combination treatments, such as gemcitabine/ docetaxel or doxorubicin plus a heart protectant, may effectively reduce individual toxicities while boosting responses, but these strategies are of limited utility.^12^, ^13^ The precise pathways contributing to occurrences and metastasis of osteosarcoma remain elusive and must be clarified in our search for better preventive and therapeutic measures.

Parathyroid hormone (PTH) is a polypeptide hormone secreted by parathyroid master cells. Through its action on osteoblasts and osteoclasts, via cyclic adenosine monophosphate (cAMP) and phospholipase C pathways, it serves to modulate blood calcium levels. Parathyroid hormone receptor 1 (PTHR1) belongs to the G protein–coupled cell membrane receptor family, widely distributed and of greatest import in canine and rat osteosarcoma. PTHR1 binds both PTH and PTH‐related peptide (PTHrP) and is primarily expressed in bone, kidney and cartilage. The vascular system, certain developmental organs and human MCF7 breast cancer cells also show high levels of expression.^14^, ^15^ It is encoded by 14 exon genes on chromosome 3 and plays a critical role in regulating serum and endochondral bone concentrations of calcium.^16^

**FIGURE 1:**
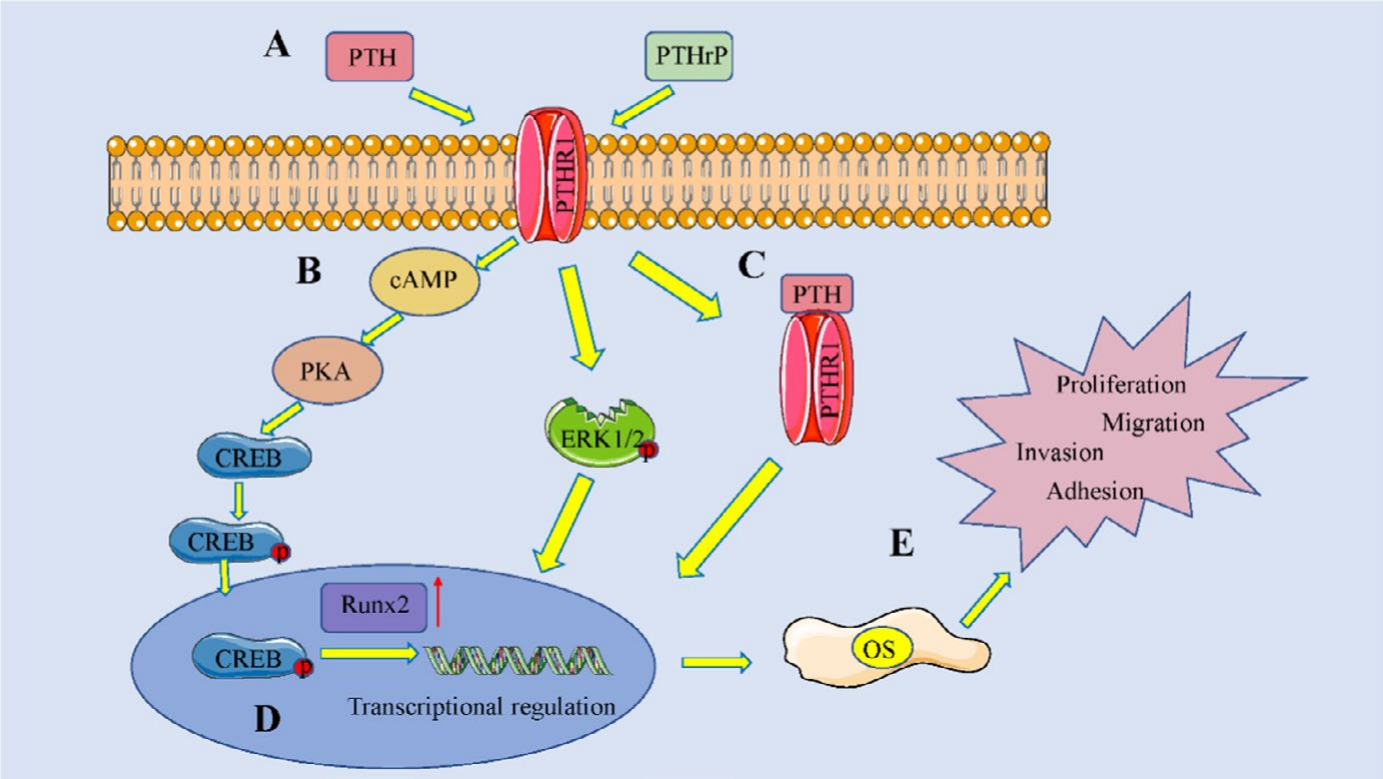
The effect of parathyroid hormone (PTH)/PTH‑related peptide (PTHrP)–dependent extracellular matrix (ECM) signalling on osteosarcoma (OS) cell functions. (A) PTHR1 activation; (B) receptor respective downstream signalling; (C) transcriptional regulation; (D) modulation of ECM‑correlated target genes; and (E) regulation of basic OS cell functions. PTHR1, PTH receptor 1; cAMP, cyclic adenosine monophosphate; CREB, cAMP response element–binding protein; and Runx‑2, runt‑related transcription factor 2

Along with other specifics (ie target cell type, molecular structure of binding ligand and homeostatic bodily conditions), PTHR1 has been implicated in a number of intracellular signalling pathways, the nature, degree and duration of which are decisive in the biologic responses induced.^17^ Biochemical and cellular responses to PTHR1 activation may thus differ according to cell type. In primary failure of eruption (PFE), clinical and radiographic characteristics are highly specific for PTHR1 effects^18^; and compared with responses in wild‐type (WT) mice, physiological responses to injected PTH ligands are acutely and severely disrupted in mice bearing the phosphorylation‐deficient (PD) PTHR1 knock‐in mutation.^19^ In both WT and PD animals, PTH administration increases the volume and trabecular thickness of vertebral and distal femoral bones, but PTHR1 phosphorylation is not a major factor in anabolic actions of PTH.^20^ PTH/PTHR1 and phytoestrogens have both performed positively in an animal model of bilateral ovariectomy.^21^ Finally, activation of PTHR1 appears to modulate diverse molecular cascades through autocrine mechanisms. These cascades are involved in a variety of processes, including hormonal feedback control, receptor desensitization and catabolism, as well as removal of hormone‐ligand complexes from the circulation.^17^, ^22^ Many studies have underscored the impact of abnormal PTHR1 expression, showing a close association with occurrences and malignant progression of osteosarcoma (Figure 1).

**FIGURE 2:**
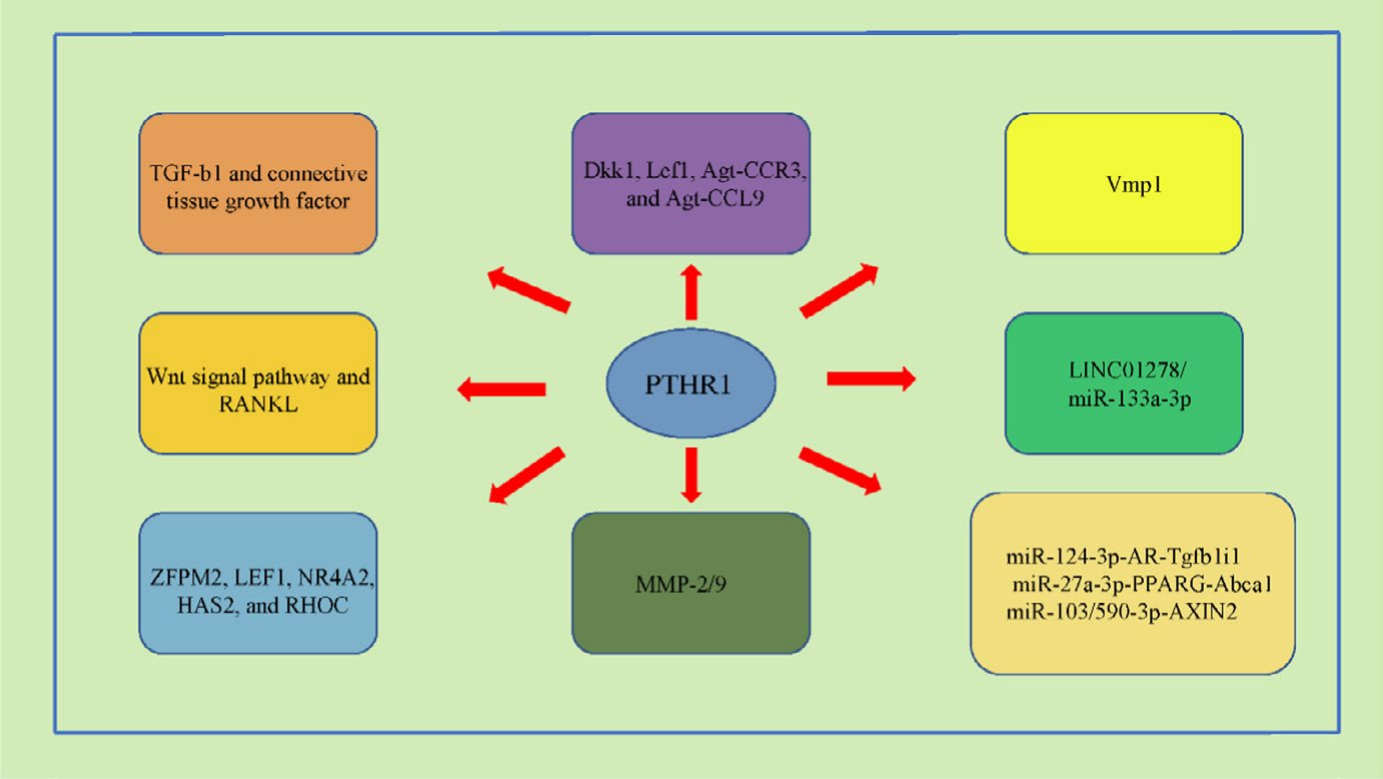
Potential mechanism and regulatory axis of PTHR1 in osteosarcoma. (A) Related to TGF‐b1 and connective tissue growth factor; (B) Wnt signal pathway and RANKL regulation; (C) potential downstream genes (ZFPM2, LEF1, NR4A2, HAS2 and RHOC); (D) potential downstream genes (DKK1, LEF1, Agt‐CCR3 and Agt‐CCL9); (E) MMP‐2/9 regulation; (F) VMP1 regulation; (G) regulated by LINC01278/ miR‐133a‐3p; and (H) potential regulatory axis (miR‐124‐3p‐AR‐Tgfb1i1, miR‐27a‐3p‐PPARG‐Abca1 and miR‐103/590‐3p‐AXIN2). RANKL, TNF superfamily member 11; ZFPM2, zinc finger protein, FOG family member 2; LEF1, lymphoid enhancer–binding factor 1; NR4A2, nuclear receptor subfamily 4 group A member 2; HAS2, hyaluronan synthase 2; RHOC, ras homolog family member C; DKK1, dickkopf WNT signalling pathway inhibitor 1; MMP‐2/9, matrix metallopeptidase 2/9; and VMP1, vacuole membrane protein 1

In this review, we have summarized present knowledge surrounding regulatory and functional aspects of PTHR1 in osteosarcoma. We have also probed the clinical implications of altering PTHR1 expression and the latest therapeutic strategies for targeting PTHR1 in this setting (Table 2, Figure 2).

**TABLE 2:** The biological function of PTHR1 in osteosarcoma

Researchers	Expression	Functional role	Related gene	References
Yang et al	Up‐regulated	Cell proliferation and invasion	TGF‐b1 and connective tissue growth factor	^32^
Ho et al	Up‐regulated	Cell invasion, growth and tumour differentiation	Wnt signal pathway and RANKL	^44^
Guan et al	/	/	ZFPM2, LEF1, NR4A2, HAS2 and RHOC	^43^
Li et al	/	/	Dkk1, Lef1, Agt‐CCR3 and Agt‐CCL9	^17^
Li et al	Up‐regulated	Cell proliferation, invasion and tumorigenesis	MMP‐2/9 and Vmp1	^40^
Li et al	Up‐regulated	Cell proliferation and tumour metastasis	MMP‐2/9	^39^
Li et al	/	/	miR‐124‐3p‐AR‐Tgfb1i1, miR‐27a‐3p‐PPARG‐Abca1, and miR‐103/590‐3p‐AXIN2	^42^
Wen et al		Cell proliferation and tumour metastasis	MMP‐2/9	^41^
Qu et al	Up‐regulated	Cell proliferation and apoptosis	LINC01278/ miR‐133a‐3p	^31^
Al‐Khan et al	Up‐regulated	Prognostic role	/	^14^

## 2 BIOLOGY OF PTHR1

PTHR1 is a class B G protein–coupled receptor that binds PTH and PTHrP. Although present in other tissues, it is primarily expressed in bone, kidney and cartilage.^23^, ^24^ Despite their shared signalling mechanisms, the biologic functions of PTH and PTHrP are actually quite different.^25^, ^26^ PTH modulates serum calcium through endocrine effects on bone and kidney cells, whereas PTHrP is a paracrine modulator of cell proliferation and differentiation at developmental sites, such as bony growth plates.^22^ The biologic responses induced by PTHR1 activation generally reflect the nature, intensity and duration of signalling, in conjunction with other variables (ie target cell type, ligand structure and primary homeostatic conditions). Activation of PTHR1 thus triggers unique biochemical and cellular responses, depending on cell type.

In osteoblasts and chondrocytes, PTHR1 activation modulates proliferative and apoptotic efficiency and contributes to production of various signalling factors involved in bone and cartilage metabolism.^22^ In renal tubules, PTHR1 activation modulates transmembrane transport of mineral ions through expression levels and functional activities of related proteins. The global response to PTHR1 activation is modulated by processes at several levels, including intracellular routes for receptor desensitization, systemic feedback loops for hormonal release, and steps in the destruction and metabolic clearance of peptide hormones from the circulation. Despite the many mechanisms that modulate PTHR1 activity, maladjustments may occur, imposing serious physiological consequences.^17^, ^27^, ^28^

## 3 EXTENT AND CLINICAL RELEVANCE OF PTHR1 EXPRESSION IN OSTEOSARCOMA

In patients with osteosarcoma, PTHR1 overexpression has been linked to greater risk of metastasis and a poor prognosis. Using quantitative reverse transcription‐polymerase chain reaction (QRT‐PCR), Qu et al have recorded dramatically higher levels of PTHR1 expression in a 40‐patient sampling of osteosarcomas (vs normal control tissues), culminating in adverse clinical outcome.^29^ Levels of PTHR1 mRNA expressed in metastases or recurrences of osteosarcoma have also proven much higher than those in primary tumours, conferring more aggressive phenotypes and microenvironments more conducive to malignancy.^30^ A canine model of osteosarcoma constructed by Al‐khan and colleagues has demonstrated a poorer prognosis for dogs showing intensified PTHR1 expression.^31^ Besides, PTHrP (1‐40) stimulates calcium uptake which transporters TRPV6 and CaBP‐D9k via PTHR1 receptor and PKC α/β signaling pathway in rat enterocytes.^32^As a whole, these findings indicate the important influence of PTHR1 on progression of osteosarcoma and its utility in predicting patient prognosis.

## 4 REGULATORY MECHANISMS OF PTHR1 IN OSTEOSARCOMA

Various sources have confirmed that PTHR1 actually modulates malignant progression of osteosarcoma through certain mechanisms involving matrix metalloproteinases, non‐coding RNA and genetic foci. The precise regulatory mechanisms and their potential for new therapeutic targets must be further studied and fully explored.

### 4.1 Matrix metalloproteinases and extracellular matrix regulation by PTHR1

The family of matrix metalloproteinases (MMPs), which variably relies on metal (ie, zinc) and calcium ions as cofactors, is now comprised of at least 26 different members assigned to five major substrate‐defined categories as follows: interstitial collagen enzyme, gelatin enzymes, stromelysin, metalloproteinases and other enzymes. Given their importance in tissue remodelling and organ development, abnormal MMP expression (especially MMP‐2, MMP‐9 and MMP‐14) is seen in a host of diseases, from autoimmune disorders to cancers, impacting tumour and immune‐cell microenvironments.^33^, ^34^, ^35^, ^36^

PTHR1 likely stimulates malignant progression of osteosarcoma by regulating MMP expression. In a past study of metastatic human osteosarcoma cells, quercetin clearly diminished expression of PTHR1 mRNA, thus attenuating expression levels of MMP‐2 and MMP‐9 mRNA and ultimately reducing cellular invasion, adhesion, proliferation and migration.^37^ Li and colleagues have similarly witnessed attenuation of PTHR1 mRNA and protein expression in osteosarcoma cells incubated with β‐alanine, indicating its positive relation with tumour invasion and metastasis^38^; and upon incubating Saos‐2 and U2OS human osteosarcoma cell lines in β‐alanine, Wen et al found expression levels of MMP‐2 and MMP‐9 mRNA notably reduced, whereas tissue inhibitors of metalloproteinase (TIMP)‐1 and TIMP‐2 were expressed at high levels. Osteosarcoma cells treated in mangiferin (a xanthone) have also shown substantial attenuation of PTHR1 mRNA and protein expression, along with growth inhibition and induction of apoptosis.^39^ Nonetheless, the interplay of PTHR1 and MMP expression levels in progression of osteosarcoma requires greater scrutiny.

The extracellular matrix (ECM) is an extensive network of cellular secretions, composed of collagens, elastin, proteoglycans (PGs), glycosaminoglycans (GAGs), fibronectins and laminins. Relative to physiological bone, the ECM of osteosarcoma is extensively altered. At least one prior study has addressed the role of ECM role in regulating osteosarcoma cells in vivo and in vitro.^40^ Fibroblast growth factor‐2 is known to modulate migration of MG‐63 osteosarcoma cells by regulating expression levels of ECM‐associated proteoglycans.^41^ Downstream signalling pathways of the ECM components are distinctive^42^, ^43^ and have received growing attention for their interconnected mechanisms of carcinogenesis in osteosarcoma. Broader study is essential in this area to assess their potential as clinical biomarkers and therapeutic targets.

The microenvironment, including bone, stroma, vascular elements and immune cells, is also critical in regulating the growth and metastasis of osteosarcoma.^44^ Mesenchymal stem cells (MSCs) within the microenvironment of bone are paramount in promoting tumour metastasis, and many signalling pathways, such as PI3K/Akt, Wnt/β‐Catenin, MAPK/ERK, Hedgehog and Notch, facilitate the transition of MSCs to osteosarcoma cells. In animal models, MSCs clearly contribute to pulmonary metastasis.^45^ Immunotherapy has gained prominence as a clinical treatment, showing promise in patients with lung cancer, melanoma, and oesophageal cancer. Tumour immune cells are varied, consisting of mesenchymal cells, tumour‐infiltrating immune cells (TIICs), endothelial cells, ECM molecules and inflammatory mediators. Immune scores are associated with overall survival and gauge immune‐related risk in patients with osteosarcoma.^46^

### 4.2 Non‐coding RNA and key genes implicated in PTHR1 regulation

Non‐coding RNA (ncRNA) chiefly includes microRNA (miRNA), long non‐coding RNA (lncRNA) and circular RNA (circRNA). Because the assumed region is a complementary base pair to the seed sequence of miRNA, located at its 3' untranslated region (UTR), PTHR1 may be influenced by regulating miRNAs. According to Li et al, *PTHR1* may exert important influence on progression of osteosarcoma by activating miRNA genes miR‐124‐3‐p‐*AR‐Tgfb1i1*, miR‐a‐3p‐*PPARG‐27 Abca1* and miR‐103/590‐3p‐*AXIN2*.^47^ Qu et al have also noted that in osteosarcoma, LINC01278 is a competing endogenous RNA of PTHR1 (by sponging miR‐133a‐3p) and a likely point of tumour inhibition in osteosarcoma.^29^

The vastness of bioinformatics applications has allowed researchers to trace many potential downstream targets of PTHR1. In particular, *NR4A2*, *ZFPM2*, *RHOC*, *LEF1* and *HAS2* may be potential targets of *PTHR1* in osteosarcoma^48^; and the pro‐malignant effects of *PTHR1* are apparently mediated through Wnt, angiogenesis and fever pathways, modulating expression of pivotal enriched genes (*Dkk1*, *Lef1*, *AGT‐CCR3* and *AGT‐CCL9*).^15^ *PTHR1* may thus regulate *AGT‐CCL9* in osteosarcoma cells, impacting cellular viability, apoptosis, migration, invasion and colony formation.^49^

## 5 FUNCTION OF PTHR1 IN OSTEOSARCOMA

As a crucial oncogenic gene, oncogenic properties of *PTHR1* pertaining to osteosarcoma have been adequately chronicled. In this section, we describe the functions of *PTHR1* as a pro‐oncogenic gene in this setting, serving to regulate PTHR1 expression.

### 5.1 Promoting cellular proliferation and growth

In the absence of added exogenous PTHrP, overexpression of PHTR1 intensifies cellular proliferation, motility and Matrigel invasion, as presumptive autocrine effects. PTHR1 overexpression is also associated with deferred osteoblastic differentiation and up‐regulation of genes involved in ECM production, inclusive of TGF‐b1 and connective tissue growth factor.^30^ Ho and colleagues have used shRNA to diminish PTHR1 expression, observing a mild hindrance of cellular proliferation in vitro but markedly diminished collagen invasion and reduced expression of RANK ligand (RANKL). Although in vivo administration of PTH (1‐34) has no proliferative effect on osteosarcoma cells, knockdown of PTHR1 yields remarkable growth inhibition and intensifies tumour differentiation/mineralization.^50^ In a study by Wen et al, mangiferin treatment served to greatly reduce expression of PTHR1 mRNA and protein in osteosarcoma cells, curbing their viability, proliferation, invasion, adhesion, and migration, and triggering apoptosis.^39^ Al‐Khan et al have investigated expression levels of PTHR1 and PTHrP in canine osteosarcoma tissues as a means of prognostication. They found that overexpression of PTHR1 antigen correlated with poorer outcome, supporting its use as prognostic index.^31^

### 5.2 Promoting cellular invasion and metastasis

Li et al have shown that β‐alanine inhibition of PTHR1 expression diminishes proliferation, invasion, migration and tumorigenesis in U2OS cells, signifying a positive relation between PTHR1 expression and tumour invasion/metastasis.^38^ Quercetin‐inhibited proliferation and inhibition of osteosarcoma are otherwise enhanced by knockdown of PTHR1, underscoring its importance in this setting.^37^

## 6 THERAPEUTIC STRATEGIES BASED ON PTHR1 IN OSTEOSARCOMA

As shown by Qu et al, LINC01278 (an lncRNA affiliate) promotes cellular proliferation and limits apoptosis in osteosarcoma cells. Mechanistic studies further indicate that LINC01278 is a competing endogenous RNA of PTHR1 (by sponging miR‐133a‐3p) and a likely point of tumour inhibition. Down‐regulation of PTHR1 serves to restore the inhibitory impact of miR‐133a‐3p. This carcinogenic effect of LINC01278, as a consequence of miR‐133a‐3p/PTHR1 signalling, represents a viable opportunity for therapeutic targeting.^29^

## 7 CONCLUSION AND FUTURE PERSPECTIVES

Patients with advanced osteosarcoma are currently confined to systemic chemotherapy as treatment, although the limitations of present‐day regimens have become increasingly evident.^51^, ^52^ Finding new treatments is therefore a matter of urgency. The mTOR pathway inhibitor, rapamycin, mitigates mRNA translation and inhibits metastasis of osteosarcoma cells,^53^, ^54^ but prospects beyond this are seriously lacking. Although targeted therapy is the new paradigm in treating advanced malignant tumours, the research to date on targeted treatments for osteosarcoma remains insufficient. More studies and drug testing are needed to identify better and safer agents.

The G protein–coupled receptor PTHR1 is highly expressed in cartilage, kidney, bone, and other vascular and developmental tissues. It is encoded by 14 exon genes on chromosome 3 and is critical in regulating calcium concentrations of serum and endochondral bone. A number of intracellular signalling pathways are linked to PTHR1, the nature, intensity and duration of signalling determining subsequent biologic responses. There are ancillary variables as well, including target cell type, molecular structure of binding ligand and homeostatic conditions.^16^, ^20^, ^55^ Biochemical and cellular responses thus differ according to cell type.^24^, ^56^, ^57^, ^58^

In osteoblasts and chondrocytes, activation of PTHR1 modulates proliferation, apoptosis and production of assorted signal transduction factors involved metabolism of bone and cartilage. In renal tubules, PTHR1 activation modulates the transmembrane transport of mineral ions by regulating expression levels and functions of related proteins. Through autocrine mechanisms, PTHR1 activation also modulates diverse molecular cascades involved in receptor desensitization, hormonal feedback loops, catabolism and removal of hormone‐ligand complexes from circulation.

Clinical data on patients with osteosarcoma indicate that PTHR1 overexpression carries greater risk of metastasis and a poor prognosis. Investigational studies have likewise shown that PTHR1 plays an important pro‐oncogenic role in tumour growth and distant metastasis, both in vitro and in vivo. Hence, a new therapeutic strategy for osteosarcoma, aimed at inhibiting PTHR1 expression or function, seems quite feasible. This may involve specific mechanisms under genetic and non‐genetic control, using epigenetic drugs and natural compounds known to target PTHR1 in vitro. Because specific mechanisms of PTHR1 activity have yet to be fully delineated in the context of osteosarcoma, our research efforts must intensify going forward, propelling routine therapeutics to new and more acceptable heights.

## CONFLICT OF INTEREST

The authors declare no competing interests.

## AUTHOR CONTRIBUTION

Original draft preparation,allocation, revision, supplement and edition, Chaonan Sun and Shenglong Li. All authors have read and agreed to the published version of the manuscript.
